# Ion Interference Reduces the Uptake and Accumulation of Magnesium Ions in Tea Plants (*Camellia sinensis*)

**DOI:** 10.3390/plants14050643

**Published:** 2025-02-20

**Authors:** Jishuang Zou, Lihe Shi, Weiting Cheng, Yulin Wang, Yankun Liao, Junbin Gu, Tingting Wang, Qi Zhang, Jianghua Ye, Haibin Wang, Xiaoli Jia

**Affiliations:** 1 College of Tea and Food, Wuyi University, Wuyishan 354300, China; 2 College of Life Science, Longyan University, Longyan 364012, China; 3 College of Horticulture, Fujian Agriculture and Forestry University, Fuzhou 350002, China

**Keywords:** tea plant (*Camellia sinensis*), magnesium ion, absorption rate, gene expression

## Abstract

Magnesium (Mg) ions play a crucial role in the growth and development of tea plants (*Camellia sinensis*). In this study, the ion kinetic method was used to analyze the effect of ions from various elements on the Mg ion uptake rate in two tea plant varieties (Rougui and Shuixian). Additionally, Mg ion content and the expression intensity of *CsMGT5* gene in the tea plant’s root system were measured to further understand how different elemental ions affect Mg ion uptake and accumulation. The results revealed that while the trends in the effects of different elements on Mg ion uptake were similar in both Rougui and Shuixian roots, the magnitude of these effects was lower in Rougui and higher in Shuixian. In the presence of only Mg ions in the solution, the tea plant’s root system exhibited the highest intensity of *CsMGT5* gene expression, the fastest uptake rate of Mg ion, and the highest Mg content. Conversely, the presence of nitrogen, phosphorus, and potassium ions alone reduced *CsMGT5* gene expression, Mg ion uptake rate, and Mg content in the tea plant’s root system. However, differences in the impact of these three elements on Mg ion uptake and accumulation were not statistically significant. In addition, with the increase in the types of added ions, the Mg ion uptake rate by tea plants gradually declined, indicating a decreasing demand, with Mg accumulation showing a downward trend. Statistical analysis of correlations showed that *CsMGT5* gene expression in the tea plant’s root system positively regulated the maximum uptake rate of Mg ion (I_max_ value, 0.94 **). The I_max_ value negatively regulated Mg ion content in solution (C_min_ value, −0.94 **), and the C_min_ value negatively regulated Mg ion content in the tea plant’s root system (−0.95 **). In conclusion, the presence of different elemental ions significantly influenced the uptake and accumulation of Mg ions in tea plants, with the magnitude of this effect intensifying as the number of elemental types increased. A positive correlation was observed between the capacity for Mg ion uptake and accumulation capacity in the tea plant’s root system and the expression intensity of the *CsMGT5* gene within the root system. This study offers valuable insights and serves as an important reference for leveraging Mg to regulate tea plant growth in practical agricultural applications.

## 1. Introduction

Magnesium (Mg) plays a crucial role in plant growth, and plant tissues require about 1.5~3.5 mg/g of Mg ions for optimal plant growth [1]. Mg affects physiological and biochemical processes in plants, such as chlorophyll synthesis, photosynthesis, and protein synthesis which, in turn, affects plant growth and development and yield formation [2,3,4]. Furthermore, Mg is an activator of a high number of enzymes in plant cells [5]. Mg deficiency leads to chlorisis and necrosis of plant leaves, reduced carbohydrate fixation in leaves, uneven distribution, slowing plant growth, and even death [6,7].

Tea plants (*Camellia sinensis*) are economically important plants that are mainly harvested for their young shoots and leaves [8]. Mg is important for the growth, development and regulation of the physiological metabolism of the tea plant [9,10,11]. However, the uptake process of Mg ions in plants can also be disturbed by ions of different elements which, in turn, alters the plant’s ability to absorb and accumulate Mg ions. Xie et al. [12] found that there is an antagonistic effect between K and Mg, and the presence of K ions reduces the plant’s ability to take up Mg. Qu et al. [13] found that Mg significantly affected the uptake of other elements, particularly K and Ca (calcium), in *Solanum lycopersicum* L. and *Cucumis sativus* L., and that the uptake of K and Ca decreased in both plants as Mg concentrations increased. It has also been shown that hydrogen (H), aluminum (Al), and Mn ions can interfere with the uptake of Mg ions by the plant root system, and reduce the accumulation and transport capacity of Mg ions in the plant root system, leading to Mg deficiency in plants [14]. K and ammonium (NH_4_) ions can also interfere with the uptake of Mg ions in plants, reducing the plant’s ability to absorb and transport Mg ions, especially in acidic soils [15]. It can be seen that plants may be interfered with by a variety of elements during the uptake of Mg ions which, in turn, alters the plant’s ability to absorb and accumulate Mg ions.

The root system is the main tissue for nutrient uptake in plants, and the uptake and accumulation of nutrient elements by the root system is essential for plant growth [16]. The rate of nutrient uptake by the plant root system is closely related to accumulation capacity, and the higher the rate of uptake, the more favorable the accumulation of nutrient elements [17]. Elemental uptake kinetics is the study of the rate at which ions are taken up from solution by the plant root system, and the data obtained, when simulated by mathematical models, can be used to analyze the effects of environmental factors on plant ion uptake which, in turn, can be beneficial in balancing plant nutrient supply [18]. Secondly, The ability of tea plants to absorb and accumulate Mg ions is closely related to the expression of the *CsMGTs* gene family, and different *CsMGTs* genes regulate the uptake and transport of Mg ions in different tea plant tissues [19]. Among them, the *CsMGT5* gene is a key gene mediating the uptake of Mg ions in the tea plant’s root system, and its expression intensity directly affects the uptake and accumulation capacity of Mg ions in tea plant [20]. Hypothetically, different elements may interfere with the uptake of Mg ions by the tea plant which, in turn, affects the accumulation of Mg in the root system of the tea plant. Accordingly, this study took different varieties of tea plants as the research object, and analyzed the effect of Mg alone and coexisting with different elements on the absorption rate of Mg ions in tea plants by ion uptake kinetic method, to explore the effect of different elements on the absorption rate of Mg ions in tea plants. Meanwhile, tea plant roots were collected to determine the Mg content of tea plant roots and the expression intensity of *CsMGT5*, a key gene for Mg transport. On this basis, the relationship between the expression intensity of the key gene *CsMGT5*, Mg content and the uptake rate of Mg ion in the tea plant’s root system under different elements was further analyzed, with a view to providing certain references for the Mg regulation of tea plant growth and development.

## 2. Results and Discussion

### 2.1. Analysis of Simulation Equations for the Kinetics of Mg Ion Uptake in Tea Plant Root System

In this study, the influence of different elements on Mg ion uptake in the tea plant’s root system was analyzed using ion uptake kinetic method, and the kinetic equation for Mg ion uptake in the tea plant’s root system was constructed. Simulation analysis of equations for Mg ion uptake by roots of different tea plant varieties showed that (Table 1), for the R^2^ values of simulation equations for Mg ion uptake by Rougui (*Camellia sinensis*) root system, repetition 1 ranged from 0.9466 to 0.9744, repetition 2 ranged from 0.9320 to 0.9799, and repetition 3 ranged from 0.9454 to 0.9710, while for the R^2^ values of the simulation equation for Mg uptake in Shuixian (*Camellia sinensis*) roots, replicate 1 ranged from 0.9271 to 0.9961, replicate 2 ranged from 0.9209 to 0.9878, and replicate 3 ranged from 0.9497 to 0.9975. It can be seen that the simulation equations for the uptake of Mg ions by the roots of different tea plant varieties were well-fitted and suitable for further analysis.

### 2.2. Effect of Different Elements on the Rate of Mg Ion Uptake in the Tea Plant’s Root System (Camellia sinensis)

**Figure 1:**
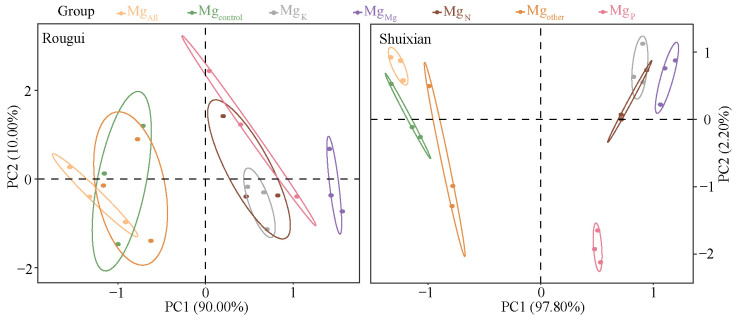
Principal component analysis of the kinetic parameters of Mg ion uptake in the tea plant’s root system under different elements. Note: Mg_control_ is tea plant seedlings transplanted to complete nutrient solution without starvation treatment; Mg_All_ is tea plant seedlings transplanted to complete nutrient solution after starvation treatment; Mg_N_, Mg_P_, and Mg_K_ are starvation-treated tea seedlings transplanted into Mg ion culture medium containing only N, P, or K, respectively; and Mg_other_ is starvation-treated tea seedlings transplanted to complete nutrient solution without N, P, and K; Mg_Mg_ is starvation-treated tea plant seedlings transplanted to a culture solution containing only Mg ions.

**Figure 2:**
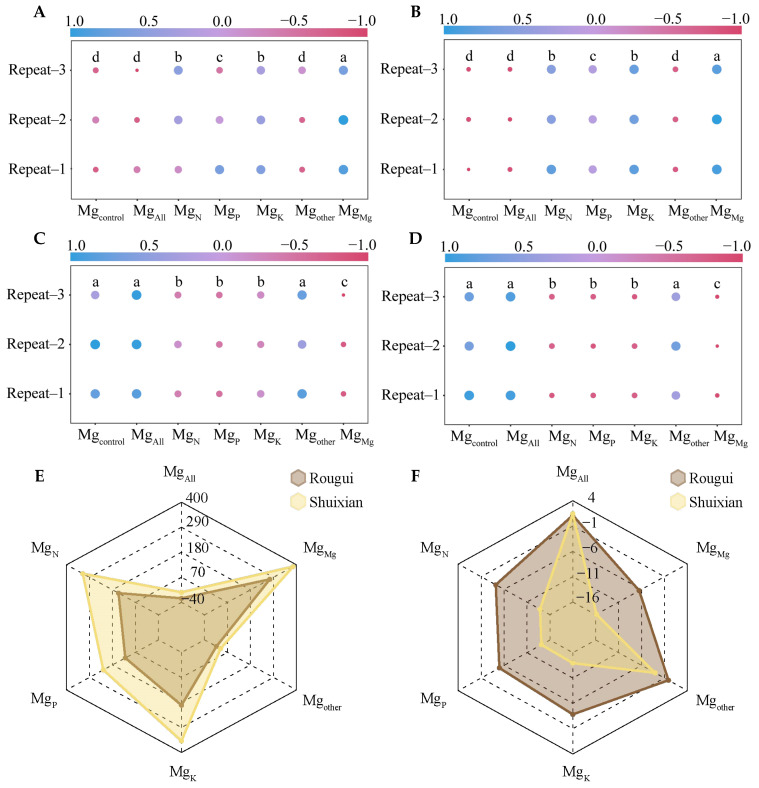
Analysis of the kinetic parameters of Mg ion uptake in the tea plant’s root system under different elements. Note: Mg_control_ is tea plant seedlings transplanted to complete nutrient solution without starvation treatment; Mg_All_ is tea plant seedlings transplanted to complete nutrient solution after starvation treatment; Mg_N_, Mg_P_, and Mg_K_ are starvation-treated tea seedlings transplanted into Mg ion culture medium containing only N, P, or K, respectively; and Mg_other_ is starvation-treated tea seedlings transplanted to complete nutrient solution without N, P, and K; Mg_Mg_ is starvation-treated tea plant seedlings transplanted to a culture solution containing only Mg ions; (**A**) maximum Mg ion uptake rate (I_max_) of Rougui root system; (**B**) maximum Mg ion uptake rate (I_max_) of Shuixian root system; (**C**) Mg ion concentration in the solution when the Mg ion uptake rate of Rougui root system is zero (C_min_ value); (**D**) Mg ion concentration in the solution when the Mg ion uptake rate of Shuixian root system is zero (C_min_); (**E**) difference between I_max_ value of Rougui or Shuixian root system under different elements (▲I_max_), using _Mgcontrol_ as control; (**F**) difference between C_min_ value of Rougui or Shuixian culture solution under different elements (▲C_min_) using Mg_control_ as control; different lowercase letters indicate that the differences between different treatments reached the *p* < 0.05 level.

In this study, the ion kinetic method was used to analyze the effects of different elements on the absorption of Mg ions in the root system, to obtain the two kinetic parameters of Mg ion uptake, namely the maximum absorption rate of Mg ions in the tea plant’s root system (I_max_ value) and the concentration of Mg ions in the solution when the rate of Mg ions absorbed by the tea plant’s root system was zero (C_min_ value). Principal component analysis with I_max_ and C_min_ values under different elemental treatments found (Figure 1) that for Rougui, the differentiation between the seven different elemental treatments was weak, and there was some crossover between treatments, whereas for Shuixian, the differentiation between the seven different elemental treatments was relatively good. Further analysis of the effect of different elements on I_max_ values for Mg ion uptake by tea plant roots revealed (Figure 2A) that the trend in I_max_ values of Mg ion uptake by Rougui roots under different elements was Mg_Mg_ > Mg_K_ > Mg_N_ > Mg_P_ > Mg_other_ > Mg_All_ > Mg_control_. Among them, there was no significant difference between Mg_All_, Mg_control_, and Mg_other_, and there was no significant difference between Mg_N_ and Mg_K_; however, Mg_Mg_ was significantly greater than Mg_N_ and Mg_K_, Mg_N_ and Mg_K_ were significantly greater than Mg_P_, and Mg_P_ was significantly greater than Mg_All_, Mg_control_, and Mg_other_. Analysis of I_max_ values for Mg ion uptake by Shuixian roots showed (Figure 2B) that I_max_ values under different elements was Mg_Mg_ > Mg_K_ > Mg_N_ > Mg_P_ > Mg_other_ > Mg_All_ > Mg_control._ Among them, there was no significant difference between Mg_All_, Mg_control_, and Mg_other_, and there was no significant difference between Mg_N_ and Mg_K_; however, Mg_Mg_ was significantly greater than Mg_N_ and Mg_K_, Mg_N_ and Mg_K_ were significantly greater than Mg_P_, and Mg_P_ was significantly greater than Mg_All_, Mg_control_, and Mg_other_. Antagonism between ions affects the uptake of ions by plants, which is usually faster for monovalent ions than for divalent ions [21]. Moreover, the greater the number and type of ions, the stronger competition and antagonism between ions [22]. Therefore, when there were only Mg ions in the solution, there was no antagonism between the ions and the rate of uptake of Mg ions by the tea plant reached a maximum. When other ions were present in the solution, the rate of uptake of Mg ions by the tea plant decreased with increasing ionic types.

**Table 1:** Equation of Mg ion concentration in tea plant culture solution with time under different nutrient element treatments.

	Treatment	The Simulation Equation of Mg Ion Concentration Change					
		Repeat 1		Repeat 2		Repeat 3	
Rougui	Mg_control_	C = 0.0193t^2^ − 0.8309t + 40.243	R^2^ = 0.9744	C = 0.0358t^2^ − 1.1029t + 40.817	R^2^ = 0.9799	C = 0.0150t^2^ − 0.8598t + 41.448	R^2^ = 0.9454
	Mg_All_	C = 0.0285t^2^ − 1.0544t + 41.384	R^2^ = 0.9536	C = 0.0201t^2^ − 0.8050t + 40.289	R^2^ = 0.9719	C = 0.0144t^2^ − 0.6315t + 39.163	R^2^ = 0.9710
	Mg_N_	C = 0.0300t^2^ − 1.0287t + 35.260	R^2^ = 0.9466	C = 0.0528t^2^ − 1.3075t + 35.470	R^2^ = 0.9320	C = 0.0574t^2^ − 1.4664t + 35.737	R^2^ = 0.9638
	Mg_P_	C = 0.0858t^2^ − 1.9995t + 37.448	R^2^ = 0.9507	C = 0.0695t^2^ − 1.4634t + 33.778	R^2^ = 0.9696	C = 0.0408t^2^ − 1.0898t + 33.227	R^2^ = 0.9518
	Mg_K_	C = 0.1003t^2^ − 2.2019t + 39.482	R^2^ = 0.9659	C = 0.0898t^2^ − 2.0375t + 38.301	R^2^ = 0.9572	C = 0.0781t^2^ − 1.8894t + 38.561	R^2^ = 0.9541
	Mg_other_	C = 0.0261t^2^ − 0.8686t + 38.800	R^2^ = 0.9503	C = 0.0198t^2^ − 0.8696t + 39.118	R^2^ = 0.9535	C = 0.0458t^2^ − 1.2589t + 39.884	R^2^ = 0.9548
	Mg_Mg_	C =0.0584t^2^ − 1.7316t + 37.554	R^2^ = 0.9551	C = 0.0609t^2^ − 1.8390t + 38.581	R^2^ = 0.9581	C = 0.0475t^2^ − 1.6286t + 37.780	R^2^ = 0.9692
Shuixian	Mg_control_	C = 0.0069t^2^ − 0.2439t + 39.694	R^2^ = 0.9798	C = 0.0101t^2^ − 0.5420t + 42.105	R^2^ = 0.9549	C = 0.0092t^2^ − 0.4046t + 39.962	R^2^ = 0.9946
	Mg_All_	C = 0.0166t^2^ − 0.4589t + 40.484	R^2^ = 0.9961	C = 0.0116t^2^ − 0.3326t + 40.376	R^2^ = 0.9209	C = 0.0100t^2^ − 0.4064t + 40.968	R^2^ = 0.9792
	Mg_N_	C = 0.1242t^2^ − 2.837t + 37.746	R^2^ = 0.9795	C = 0.0927t^2^ − 2.406t + 38.063	R^2^ = 0.9878	C = 0.097t^2^ − 2.4888t + 38.323	R^2^ = 0.9855
	Mg_P_	C = 0.0809t^2^ − 2.3360t + 38.843	R^2^ = 0.9874	C = 0.0718t^2^ − 2.1898t + 38.092	R^2^ = 0.9555	C = 0.0846t^2^ − 2.3411t + 38.275	R^2^ = 0.9697
	Mg_K_	C = 0.0747t^2^ − 2.2755t + 39.551	R^2^ = 0.9465	C = 0.0776t^2^ − 2.2964t + 39.252	R^2^ = 0.9382	C = 0.0655t^2^ − 2.1466t + 39.167	R^2^ = 0.9497
	Mg_other_	C = 0.0082t^2^ − 0.5152t + 39.718	R^2^ = 0.9271	C = 0.0206t^2^ − 0.6038t + 39.524	R^2^ = 0.9610	C = 0.0125t^2^ − 0.6367t + 40.097	R^2^ = 0.9975
	Mg_Mg_	C = 0.1192t^2^ − 2.9243t + 38.356	R^2^ = 0.9797	C = 0.1287t^2^ − 3.1176t + 38.757	R^2^ = 0.9878	C = 0.1056t^2^ − 2.8264t + 39.028	R^2^ = 0.9862

Note: Mg_control_ is tea plant seedlings transplanted to complete nutrient solution without starvation treatment; Mg_All_ is tea plant seedlings transplanted to complete nutrient solution after starvation treatment; Mg_N_, Mg_P_, and Mg_K_ are starvation-treated tea seedlings transplanted into Mg ion culture medium containing only N, P or K, respectively; and Mg_other_ is starvation-treated tea seedlings transplanted to complete nutrient solution without N, P, and K; Mg_Mg_ is starvation-treated tea plant seedlings transplanted to a culture solution containing only Mg ions.

When the rate of ion uptake by the plant root system is zero, it indicates that the uptake of ions by the plant root system is basically saturated, and analyzing the content of ions remaining in the solution in this state, the uptake capacity of ions by the plant can be assessed [23]. Accordingly, this study further analyzed the concentration of Mg ions in solution (C_min_ value) when the rate of Mg ion uptake by the tea plant’s root system was zero, and the results showed (Figure 2C) that, when the rate of Mg ion uptake by the root system of Rougui was zero, the trend of the change of C_min_ value was Mg_All_ > Mg_control_ > Mg_other_ > Mg_K_ > Mg_N_ > Mg_P_ > Mg_Mg_. Among them, there was no significant difference between Mg_All_, Mg_control_, and Mg_other_, and no significant difference between Mg_N_, Mg_P_, and Mg_K_; however, Mg_All_, Mg_control_, and Mg_other_ were significantly higher than Mg_N_, Mg_P_, and Mg_K_, while Mg_N_, Mg_P_, and Mg_K_ were significantly higher than Mg_Mg_. Analysis of the trend of C_min_ values when the rate of Mg ion uptake by Shuixian roots was zero showed (Figure 2D) that the trend of C_min_ values under different elements was Mg_All_ > Mg_control_ > Mg_other_ > Mg_P_ > Mg_N_ > Mg_K_ > Mg_Mg_. Among them, there was no significant difference between Mg_All_, Mg_control_, and Mg_other_, and no significant difference between Mg_N_, Mg_P_, and Mg_K_; however, Mg_All_, Mg_control_, and Mg_other_ were significantly higher than Mg_N_, Mg_P_, and Mg_K_, while Mg_N_, Mg_P_, and Mg_K_ were significantly higher than Mg_Mg_. It can be seen that when there were only Mg ions in the solution, the tea plant had a higher demand for Mg ions and a higher uptake, whereas with the increase in ionic types, the tea plant’s demand for Mg ions continued to decrease. Moreover, when nitrogen, phosphorus, and potassium ions were present alone, they reduced Mg ion uptake by tea plants, but there was no significant difference between the three.

In addition, this study found that ▲I_max_ and ▲C_min_ values under different elements were obtained using Mg_control_ as a control. Among them, the ▲I_max_ values of Mg ion uptake by Rougui roots varied between −22.13~272.40 μg/g·h, whereas those of Shuixian ranged from 4.09~384.24 μg/g·h (Figure 2E). Secondly, under different elements, the ▲C_min_ values varied between −6.50~1.12 mg/L at zero rate of Mg ion uptake by Rougui roots, while they varied between −15.82~1.42 mg/L for Shuixian (Figure 2F). Significant differences in ion uptake and accumulation capacity have been reported for different plant varieties, and such differences may be related to variety specificity [24,25]. In the present study, it was found that the trends in the effects of different elements on Mg ion uptake in different tea plant varieties were somewhat similar, but there were some differences in the intensity of the effects. The effect of different elements on the uptake of Mg ions by Rougui was low, while their effect on Shuixian was high.

### 2.3. Effects of Different Elements on the Mg Ion Content of Tea Plant Root System

**Figure 3:**
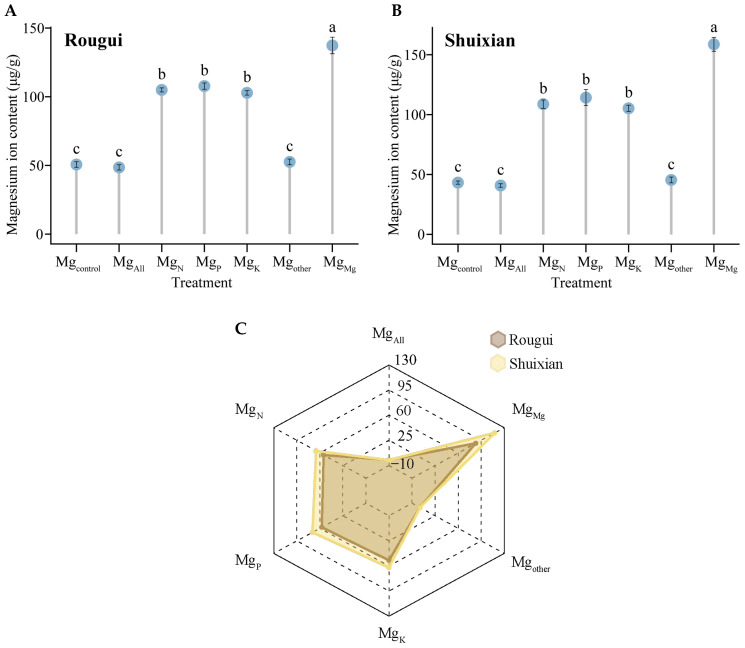
Analysis of Mg content in the tea plant’s root system under different elements. Note: Mg_control_ is tea plant seedlings transplanted to complete nutrient solution without starvation treatment; Mg_All_ is tea plant seedlings transplanted to complete nutrient solution after starvation treatment; Mg_N_, Mg_P_, and Mg_K_ are starvation-treated tea seedlings transplanted into Mg ion culture medium containing only N, P, or K, respectively; and Mg_other_ is starvation-treated tea seedlings transplanted to complete nutrient solution without N, P, and K; Mg_Mg_ is starvation-treated tea plant seedlings transplanted to a culture solution containing only Mg ions; (**A**) Mg content of Rougui root system; (**B**) Mg content of Shuixian root system; (**C**) changes in Mg content of Rougui and Shuixian root systems under different elements compared to the control; different lower-case letters indicate that the differences between treatments reached the *p* < 0.05 level.

The above analysis revealed that the uptake rate of Mg ions by the tea plant’s root system changed significantly under different nutrient mixtures, which may lead to changes in the accumulation capacity of Mg ions in the tea plant’s root system. Accordingly, the present study further determined the Mg ion content in the tea plant’s root system after treatment with different elements, and the results showed that the Mg ion content in the root system of Rougui was 50.65, 48.56, 104.96, 107.75, 102.83, 52.57, and 137.34 μg/g under seven different treatments (Figure 3A), while the Mg ion content of the root system of Shuixian were 43.20, 40.79, 108.73, 114.19, 105.28, 45.41, and 158.74 μg/g (Figure 3B). The trend of Mg ion content in Rougui and Shuixian root systems under different elements was similar, with Mg_Mg_ > Mg_P_ > Mg_N_ > Mg_K_ > Mg_other_ > Mg_control_ > Mg_All._ Secondly, it was found in this study (Figure 3) that the Mg ion content in the root systems of Rougui and Shuixian under different elements was not significantly different between Mg_All_, Mg_control_, and Mg_other_, and was not significantly different between Mg_N_, Mg_P_, and Mg_K_; however, Mg_Mg_ was significantly greater than Mg_N_, Mg_P_, and Mg_K_, while Mg_N_, Mg_P_, and Mg_K_ were significantly greater than Mg_All_, Mg_control_, and Mg_other_. It can be seen that the Mg ion content in the tea plant’s root system was closely related to the rate of Mg ion uptake by the root system. When there were only Mg ions in the solution, the tea plant had a high uptake rate and high demand for Mg ions, and when the uptake reached equilibrium, the tea plant root system had the highest Mg content. When only nitrogen, phosphorus, or potassium ions alone existed in the solution, it could reduce the rate of absorption of Mg ions by the tea plant and reduce the demand for Mg, and when the absorption reached equilibrium, the Mg content of the tea plant root system was lower, but the difference in the effect of nitrogen, phosphorus, or potassium elements on the accumulation of Mg ions in the root system of the tea plant was not significant. Secondly, with the increase in ionic types, the rate of Mg ion uptake by tea plants gradually decreased, demand decreased, and accumulation showed a decreasing trend. In addition, it was found in this study (Figure 3C) that the difference in Mg ion content in Rougui roots under different elements ranged from −2.09 to 86.69 μg/g, while that of Shuixian ranged from −2.41 to 115.54 μg/g, using Mg_control_ as a control. It can be seen that the intensity of the effect of different elements on the Mg ion content of the Rougui root system was low, while the effect on Shuixian was high.

### 2.4. Effects of Different Elements on the Expression of CsMGT5 Gene in Tea Plant Roots

**Figure 4:**
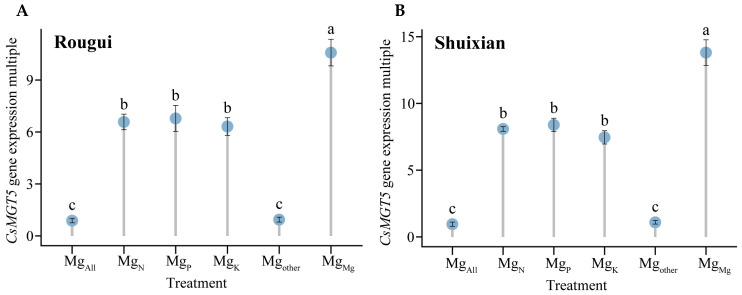
Analysis of *CsMGT5* gene expression in tea plant root system under different elements. Note: Mg_All_ is tea plant seedlings transplanted to complete nutrient solution after starvation treatment; Mg_N_, Mg_P_, and Mg_K_ are starvation-treated tea seedlings transplanted into Mg ion culture medium containing only N, P, or K, respectively; and Mg_other_ is starvation-treated tea seedlings transplanted to complete nutrient solution without N, P, and K; Mg_Mg_ is starvation-treated tea plant seedlings transplanted to a culture solution containing only Mg ions; (**A**) fold expression of *CsMGT5* gene in Rougui root system compared with the control; (**B**) fold expression of *CsMGT5* gene in Shuixian root system compared with the control; different lowercase letters indicate that the differences between treatments reached the *p* < 0.05 level.

Accordingly, this study further analyzed the intensity of *CsMGT5* gene expression in the tea plant’s root system under different elements, and the results showed that, using the *CsMGT5* gene expression under Mg_control_ treatment as the control, *CsMGT5* gene expression in the root system of Rougui under the treatments of Mg_All_, Mg_N_, Mg_P_, Mg_K_, Mg_other_, and Mg_Mg_, respectively, increased by 0.88, 6.58, 6.78, 6.32, 0.94, and 10.58-fold (Figure 4A), whereas it increased 0.95, 8.09, 8.39, 7.46, 1.09, and 13.81-fold in Shuixian root system (Figure 4B). The trends in *CsMGT5* gene expression changes in Rougui and Shuixian root systems under different elements were similar, with Mg_Mg_ > Mg_P_ > Mg_N_ > Mg_K_ > Mg_other_ > Mg_control_ > Mg_All_. Secondly, it was found in this study (Figure 4) that the expression of *CsMGT5* gene in the root systems of Rougui and Shuixian under different elements was not significantly different between Mg_All_, Mg_other_, and between Mg_N_, Mg_P_, and Mg_K_; however, Mg_Mg_ was significantly greater than Mg_N_, Mg_P_, and Mg_K_, while Mg_N_, Mg_P_, and Mg_K_ were significantly greater than Mg_All_, and Mg_other._ It can be seen that the expression intensity of *CsMGT5* gene in the tea plant’s root system was closely related to the content of Mg ions in the root system. The greater the intensity of *CsMGT5* gene expression, the faster the rate of Mg ion uptake by the tea plant’s root system, and the higher the Mg ion content in the root system. In addition, this study found that changes in *CsMGT5* gene expression under different elements ranged from 0.88 to 10.58-fold in the Rougui root system compared to Mg_control_, while it ranged from 0.95 to 13.81-fold in Shuixian (Figure 4). It can be seen that the effects of the different elements on the expression of *CsMGT5* gene in Rougui roots were relatively weak which, in turn, limited its ability to absorb and accumulate Mg ions; in contrast, the effects of these elements were more pronounced in Shuixian tea plant.

### 2.5. Statistical Analysis of Correlations

**Figure 5:**
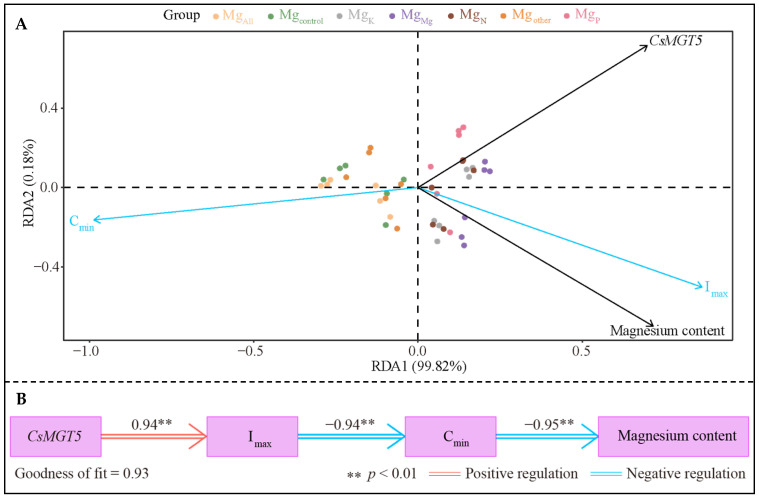
Statistical analysis of the correlation between kinetic parameters of Mg ion uptake and Mg content and *CsMGT5* gene expression in tea plant root system. Note: Mg_control_ is tea plant seedlings transplanted to complete nutrient solution without starvation treatment; Mg_All_ is tea plant seedlings transplanted to complete nutrient solution after starvation treatment; Mg_N_, Mg_P_, and Mg_K_ are starvation-treated tea seedlings transplanted into Mg ion culture medium containing only N, P, or K, respectively; and Mg_other_ is starvation-treated tea seedlings transplanted to complete nutrient solution without N, P and K; Mg_Mg_ is starvation-treated tea plant seedlings transplanted to a culture solution containing only Mg ions; (**A**) redundancy analysis between different indicators; (**B**) PLS-SEM equation construction for different indicators.

Redundancy analysis of the kinetic parameter indexes of Mg ion uptake, Mg content and *CsMGT5* gene expression in the tea plant’s root system under different elements showed that (Figure 5A), the I_max_ value of Mg ion uptake in the tea plant’s root system was significantly correlated with the Mg ion content, and gene expression of *CsMGT5*. The PLS-SEM equations for the kinetic parameter indicators of Mg ion uptake, Mg content and *CsMGT5* gene expression in tea plant root system were further constructed, and the results showed (Figure 5B) that the *CsMGT5* gene expression in the tea plant’s root system positively regulated the I_max_ value (0.94 **), the I_max_ value negatively regulated the C_min_ value (−0.94 **), and the C_min_ value negatively regulated the Mg content (−0.95 **). It is evident that the enhanced expression of *CsMGT5* gene in the tea plant’s root system could increase the absorption rate of Mg ions in the tea plant’s root system which, in turn, reduced the concentration of Mg ions in the culture broth and improved the Mg content in the tea plant’s root system.

## 3. Materials and Methods

### 3.1. Preparation of Test Material and Tea Culture Conditions

**Figure 6:**
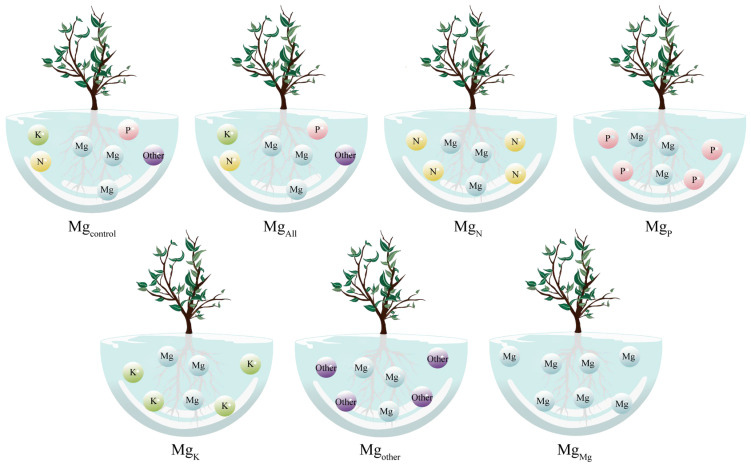
Experimental design of ion depletion. Note: Mg_control_ is tea plant seedlings transplanted to complete nutrient solution without starvation treatment; Mg_All_ is tea plant seedlings transplanted to complete nutrient solution after starvation treatment; Mg_N_, Mg_P_, and Mg_K_ are starvation-treated tea seedlings transplanted into Mg ion culture medium containing only N, P, or K, respectively; and Mg_other_ is starvation-treated tea seedlings transplanted to complete nutrient solution without N, P, and K; Mg_Mg_ is starvation-treated tea plant seedlings transplanted to a culture solution containing only Mg ions.

The tea plants selected for this study were Wuyi Rougui (*Camellia sinensis*) and Wuyi Shuixian (*Camellia sinensis*), both of which were one-year asexually propagated cuttings. The height of the tea plant seedlings was about 38 cm and their diameter of base part was 0.3 cm. Tea plant seedlings were first cleaned with deionized water to wash the root system, and then transplanted to the complete nutrient solution to cultivate the tea plant to resume normal growth. The tea plant recovery culture was carried out for 45 days. The complete nutrient solution used was formulated as 125 μmol/L KNO_3_, 187.5 μmol/L (NH_4_)_2_SO_4_, 100 μmol/L KH_2_PO_4_, 25 μmol/L K_2_SO_4_, 100 μmol/L CaCl_2_, 100 μmol/L MgSO_4_, 16 μmol/L FeSO_4_, 200 μmol/L Al_2_(SO_4_)_3_, and the pH of the culture solution was adjusted to 4.5 [26]. After 45 days of recovery culture, the tea plant seedlings were removed, the root system was rinsed using deionized water, and the tea plant seedlings were incubated in Mg-deficient complete nutrient solution for 2 days for Mg ion starvation treatment, with unstarved tea plant seedlings as the control. Ion depletion analysis was used to determine the kinetic parameters of Mg ion uptake in the tea plant’s root system seedlings [27]. Specifically, Mg ion-starved and non-starved tea plant seedlings were transplanted into plastic pots containing 2 L of culture solution, and six tea plant seedlings were transplanted into each pot. A one-way experimental design was used in this study. Seven different culture solutions (Mg_control_, Mg_All_, Mg_N_, Mg_P_, Mg_K_, Mg_other_, and Mg_Mg_) were designed to culture tea plant seedlings to analyze the effect of different elemental ions on Mg ion uptake in tea plant seedlings with three independent replicates for each treatment (Figure 6, Table S1). Specifically, Mg_control_ was the unstarved tea plant seedlings transplanted to complete nutrient solution; Mg_All_ was the starved tea plant seedlings transplanted to complete nutrient solution; Mg_N_, Mg_P_, and Mg_K_ were the starved tea plant seedlings transplanted to Mg ionized solution containing only N, P, or K (N, P, K, and Mg were supplied in the form of NH_4_NO_3_, NaH_2_PO_4_, K_2_SO_4_, and MgSO_4_, respectively); Mg_other_ was the starved tea plant seedlings transplanted to complete nutrient solution without N, P, and K; and Mg_Mg_ is the starved tea plant seedlings transplanted to culture solution containing only containing Mg ions. The contents of all other elements under different treatments were consistent with those in the complete nutrient solution.

### 3.2. Sampling of Test Material

After the tea seedlings were transplanted to the seven different culture solutions, the culture solutions were taken at 1 h intervals for a total of 15 times over a period of 0~15 h. Each sample was taken in a volume of 1 mL, with three independent replicates for each treatment. The samples taken were used to determine the concentration of Mg ions by atomic absorption spectrophotometer. During the experiment, a certain amount of deionized water was added every 30 min to maintain the volume of the culture solution basically unchanged. The experiments were carried out in an artificial climate chamber with the incubation temperature set at 25 °C, light intensity at 1500 lux, and humidity at 75%. After the ion depletion experiments were completed, the root systems of tea seedlings were collected and used to determine the fresh weight of the roots on the one hand, and the Mg content of the roots and the expression intensity of the *CsMGT5* gene on the other hand.

### 3.3. Analysis of Kinetic Parameters of Mg Ion Uptake in Tea Plant Root System

The method for the determination of Mg content in the culture solution was that 1 mL of the sample collected at a time, was fixed to 50 mL using deionized water, and passed through a 0.45 μm filter membrane. The filtrate was used to determine the Mg content by atomic absorption spectrophotometer (AA-3600, Shanghai, China) with a detection wavelength of 285.2 nm. The Mg ion uptake kinetic parameters of the tea plant root system were calculated using the method of Wang et al. [27], which mainly analyzed the maximum uptake rate of Mg ions by tea plant root system under seven different culture solution treatments, I_max_ value, and the concentration of Mg ions in the solution, C_min_ value, when the uptake rate of Mg ions by the tea plant’s root system was zero. In brief, C = c + bt + at^2^ of the quadratic equation with one unknown was constructed based on the concentration of Mg ions in solution at different time intervals obtained from the Mg ion uptake kinetics experiments, where C is the concentration of Mg ions and t is the time. The I_max_ value was calculated as I_max_ = |b| × V/FRW, where V is the volume of culture solution and FRW is the fresh weight of the root system of the tea plant seedling. The C_min_ value was calculated as C_min_ = b^2^/4a − b^2^/2a + c. Secondly, Mg_control_ was used as a control to calculate the difference between I_max_ and C_min_ values of different treatments and control values to obtain the rate of change, ▲I_max_ and ▲C_min_ values, under different elements.

### 3.4. Determination of Mg Content in the Tea Plant’s Root System

The collected root systems of tea plant seedlings were rinsed more than three times using deionized water to remove the Mg ions attached to the root systems, and then dried on 85 °C until constant weight. 0.2 g of the sample was taken, 5 mL of concentrated sulfuric acid was added for decoction, and after decoction and cold cutting, deionized water was used to finalize the sample to 50 mL, and it was passed through a 0.45 μm filter membrane [28]. The filtrate was used to determine the Mg content by atomic absorption spectrophotometer (AA-3600, Shanghai, China) with a detection wavelength of 285.2 nm.

### 3.5. Analysis of CsMGT5 Gene Expression in the Root System of Tea Seedlings

RNA from the root system of tea seedlings was extracted using TRI Reagent (Molecular Research Center, Cincinnati, OH, USA). Extracted RNA was assayed by 1% agarose gel and UV spectrophotometer (DS11+, DeNovix, Wilmington, DE, USA), respectively. Test-qualified RNA was reverse transcribed into cDNA using TaKaRa’s cDNA synthesis kit (Dalian, China). The intensity of *CsMGT5* gene expression was determined using Real-Time PCR system (StepOne Plus, Applied Biosystems, Carlsbad, CA, USA). The primer design of *CsMGT5* gene and PCR method were referred to Li et al. [20]. PCR primers for *CsMGT5* gene were CsMGT5-qF (ACTCGACAACCAGCGAAATG) and CsMGT5-qR (AGCTTCTTCGATCTGGCGTA). The PCR was programmed as 94 °C pre-denaturation for 1 min, 94 °C for 15 s, 55 °C for 30 s, 72 °C for 15 s, and 30 cycles. *CsMGT5* gene expression was calculated using the ^2−ΔΔCT^ method [29]. The *CsMGT5* gene expression in tea plant roots under Mg_control_ treatment was used as a control and set to 1. The different treatments were converted to the multiplicity of *CsMGT5* gene expression of the different treatments compared to the control, and this was used to convert *CsMGT5* gene expression multiplicity into different treatments.

### 3.6. Statistical Analysis

The raw data obtained were used for preliminary statistics and analysis using Excel 2020. Differences within treatment samples were determined using Student’s *t*-tests and F-tests, and differences between treatments were determined using Duncan’s multiple comparisons method, and differences were considered statistically significant when *p* < 0.05. Further analysis of the data and subsequent production of graphs were performed using Rstudio software (v4.2.3) [30], where the R packages used for the construction of the simulation equations for absorption dynamics were ggplot2 3.5.1 and ggpmisc 0.5.5, the R package used for the analysis of the principal components was ggbiplot 0.55, the R packages used for the bubble map were reshape2 1.4.4 and ggplot2 3.5.1, the R package used for radar map was fmsb 0.7.6, the R package used for lollipop map was ggplot2 3.5.1, the R package used for redundancy analysis was vegan 2.6.4, and the R package used for partial least squares structural equation modeling (PLS-SEM) equations was plspm 0.4.9.

## 4. Conclusions

**Figure 7:**
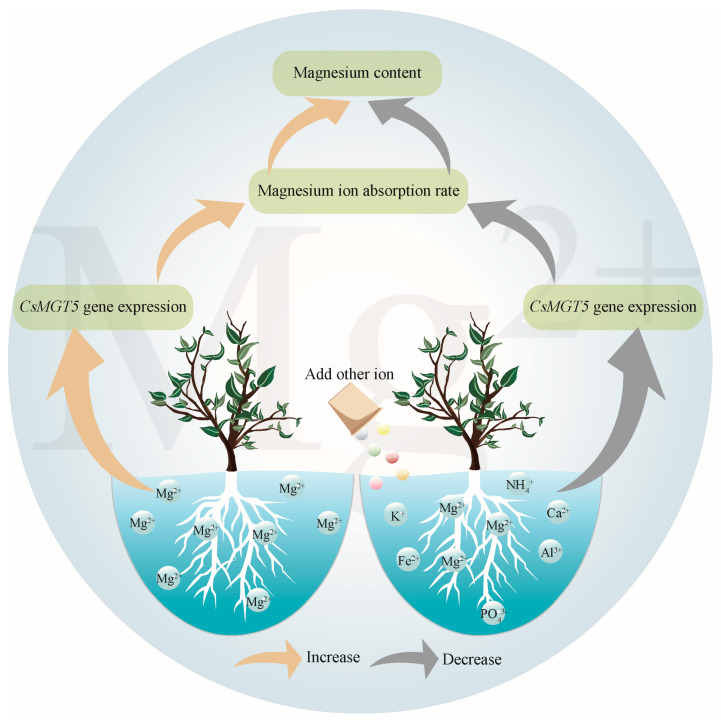
Mechanism analysis of influence of ion interference on Mg ion absorption and accumulation in tea plant roots.

In this study, the effects of N, P, and K and other elemental ions on kinetic parameters related to Mg ion uptake, Mg ion content, and the intensity of *CsMGT5* gene expression in the root system of Rougui and Shuixian tea plants were analyzed. The results showed that the effects of the different elemental ions studied on Mg ion uptake in the root system of tea plants followed a similar trend, but there were some differences in intensity. Specifically, the influence of these elements on Mg ions uptake was relatively minor in Rougui, whereas it was higher in Shuixian. Furthermore, the study found (Figure 7) that a strong correlation between Mg ion content in the tea plant’s root system and the rate of Mg ion uptake by the roots. When Mg ions were present only in the solution, the tea plant exhibited a high uptake rate and a substantial demand for Mg ions. Upon reaching equilibrium, the root system contained the highest Mg content. Conversely, when only N, P, and K ions were present in the solution, they reduced the tea plant’s Mg ions absorption rate and demand. At equilibrium, Mg content in the root system was lower, although the impact of N, P, and K on Mg ions accumulation in the root system did not significantly differ among these elements. In addition, as the number of ionic types increased, the tea plant’s Mg ion uptake rate gradually declined, with a corresponding decrease in demand and accumulation. In addition, this study also found a positive correlation between the intensity of *CsMGT5* gene expression in the tea plant’s root system and the Mg ion content within it. The greater the intensity of *CsMGT5* gene expression, the faster the uptake rate of Mg ion by the tea plant’s root system, and the higher the Mg ion content within the roots. Therefore, analyzing the effects of different elemental ions on Mg ion uptake in the root system of tea plants is of great significance in guiding the practical application of Mg regulation of tea plant growth.

## Data Availability

Data will be made available on request.

## Supplementary Materials

The following supporting information can be downloaded at: https://www.mdpi.com/article/10.3390/plants14050643/s1, Table S1: Design of ion concentration in different ion depletion experiments.
