# Supplementary material for: Ion Interference Reduces the Uptake and Accumulation of Magnesium Ions in Tea Plants (Camellia sinensis)
Source: Plants (Basel). 2025 Feb 20;14(5):643. doi: 10.3390/plants14050643 (PMC11901675; doi:10.3390/plants14050643)
Supplement: Supplementary file 1 [file plants-14-00643-s001.zip › plants-3350083-supplementary.pdf]

Supplementary data

Table S1 Design of ion concentration in different ion depletion experiments (μmol/L)

	Mg^{2+}	N^{3-}	P^{3+}	K+	Other		
					Ca^{2+}	Fe^{2+}	Al^{3+}
$Mg_{control}$	100	500	100	275	100	16	400
Mg_{All}	100	500	100	275	100	16	400
Mg_{N}	100	500					
Mg_{P}	100		100				
Mg_{K}	100			275			
Mg_{other}	100				100	16	400
Mg_{Mg}	100						
